# 1557. The Real-World Impact of Pre-Exposure Prophylaxis (PrEP) Prescription Uptake and Dispensing Status on HIV Infection Risk in the US

**DOI:** 10.1093/ofid/ofad500.1392

**Published:** 2023-11-27

**Authors:** Li Tao, Juan Yang, Woodie Zachry, Joshua Gruber, Dylan Mezzio

**Affiliations:** Gilead Sciences, Foster City, California; Gilead Sciences, Foster City, California; Gilead Sciences Inc, Foster City, California; Gilead Sciences, Foster City, California; Gilead Sciences Inc, Foster City, California

## Abstract

**Background:**

Despite effective HIV prevention options, there are continued disparities in uptake and persistence on PrEP. We assessed access to PrEP, time to dispense, and their potential relationship with HIV.

**Methods:**

Individuals receiving FTC/TDF or FTC/TAF for PrEP between Jan 2019–Feb 2023 were selected from a de-identified prescription claims database (IQVIA Longitudinal Access and Adjudication Dataset). Use of either regimen for HIV or HBV treatment or HIV PEP was excluded. Individuals were categorized into 3 groups: ≥1 PrEP claim dispensed (DISP); never had PrEP dispensed with ≥50% of claims rejected by payer (ND-R); and never had PrEP dispensed with ≥50% of claims abandoned (ND-A). A 12-month window from the index (first PrEP claim submitted) was used to assess risk of HIV infection. New HIV infection diagnoses per 100 population were calculated as no. of new HIV infections diagnosed, divided by total no. of individuals x100, in each cohort. Logistic regression was used to calculate odds ratios (OR) and 95% CIs of HIV infection by cohort and time to dispense.

**Results:**

Among 522,273 individuals, 88% were DISP, 7% were ND-R, and 5% were ND-A. Individuals in the ND-R and ND-A cohorts had a 95% and 38% higher risk of new HIV diagnosis, respectively, compared to those in the DISP cohort (Figure). For individuals with R or A claims, delayed time to dispense was significantly associated with a higher risk of acquiring HIV of ∼20%. Subgroup analysis (Table) showed that new HIV diagnoses were lowest in cisgender men who received a dispensed claim (2.1%) and highest in transgender women and transgender men in the ND-A cohort (6.1% and 4.6%, respectively), and in individuals with STIs in the ND-R and ND-A cohorts (8.1% and 5.4%).

**Figure:**
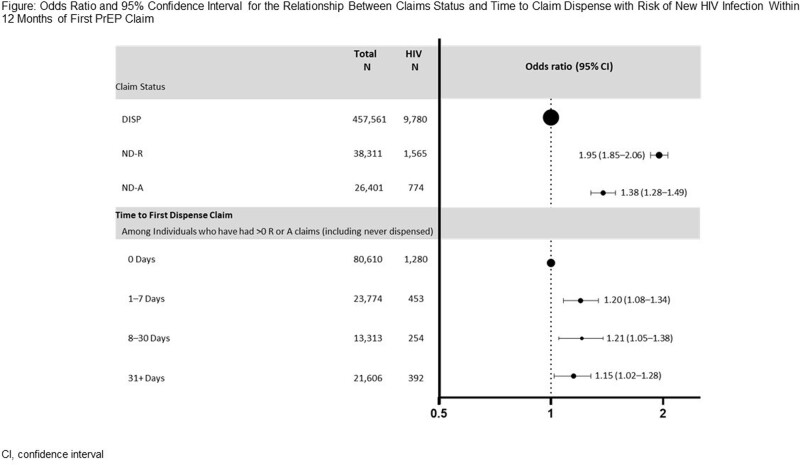

**Figure:**
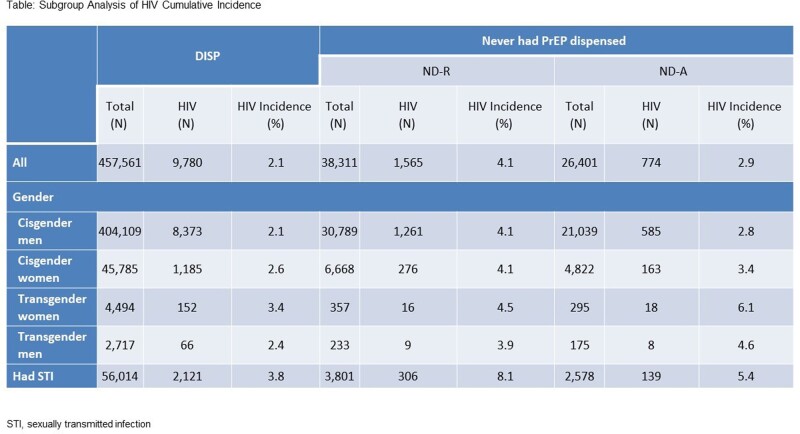

**Conclusion:**

This study is the first to find that rejected claims for PrEP were linked to a 2-fold increase in new HIV diagnoses, emphasizing the impact of timely access to PrEP on HIV prevention in real-world settings. This underscores the urgent need to remove barriers to PrEP from a prescription perspective to end HIV transmission.

**Disclosures:**

**Li Tao, MD, PhD**, Gilead Sciences, Inc: Employee|Gilead Sciences, Inc: Stocks/Bonds **Juan Yang, PhD**, Gilead Sciences, Inc: Employee|Gilead Sciences, Inc: Stocks/Bonds **Woodie Zachry, RPh, PhD**, Gilead Sciences, Inc: Employee|Gilead Sciences, Inc: Stocks/Bonds **Joshua Gruber, PhD**, Gilead Sciences, Inc: Employee|Gilead Sciences, Inc: Stocks/Bonds **Dylan Mezzio, PharmD**, Gilead Sciences, Inc: Employee|Gilead Sciences, Inc: Stocks/Bonds

